# Classification of broadband network devices using text mining technique

**DOI:** 10.1016/j.mex.2023.102346

**Published:** 2023-08-30

**Authors:** Mahasak Ketcham, Thittaporn Ganokratanaa, Nattapat Sridoung

**Affiliations:** a Department of Information Technology Management, King Mongkut's University of Technology North Bangkok, Bangkok, Thailand; b Applied Computer Science Programme, Department of Mathematics, King Mongkut's University of Technology Thonburi, Bangkok, Thailand

**Keywords:** Decision tree, naïve Bayes, Bayesian network, k-nearest neighbor, support vector machine, and deep neural network, Classification model, Text mining, Broadband network, Support vector machine, Decision support system

## Abstract

The Broadband Internet industry is highly competitive, with service providers investing heavily in network development to meet customer demands and competing on pricing. Effective cost management is crucial for profitability in this market. This work proposes a model for classifying broadband network devices based on text mining techniques applied to a device list from a leading broadband network company in Thailand. The device descriptions are used to generate a feature vector, which is then employed by a classification algorithm to categorize devices into core, access, and last mile hierarchies. Various algorithms including decision tree, naïve Bayes, Bayesian network, k-nearest neighbor, support vector machine, and deep neural network are compared, with support vector machine achieving the highest accuracy of 90.35%. The results are visualized to provide insights into network hierarchy, device replacement dates, and budget requirements, enabling support for cost management, budget planning, maintenance, and investment decision-making. The methodology outline includes,

• Obtaining a device list from a major broadband network company and extracting device descriptions through text mining and generating a feature vector.
• Using a support vector machine for classification and comparing algorithm performances.
• Visualizing the results for actionable insights in cost management, budget planning, and investment decisions.

## Graphical abstract

**Figure:**
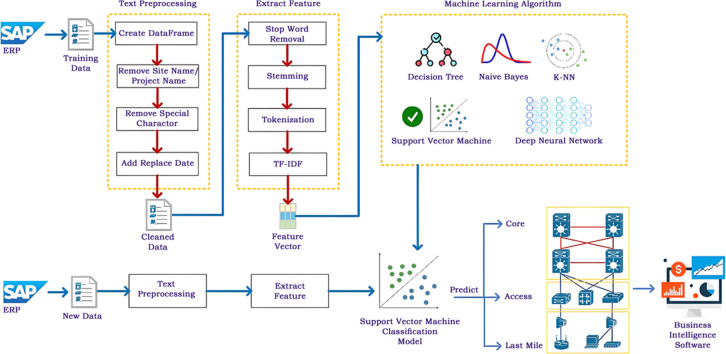

Specifications Table

**Table:** 

Subject area:	Computer Science
More specific subject area:	Text Mining, Classifier
Name of your method:	Decision tree, naïve Bayes, Bayesian network, k-nearest neighbor, support vector machine, and deep neural network
Name and reference of original method:	Decision tree Quinlan, J. R. (1986). Induction of decision trees. Machine learning, 1, 81–106. naïve Bayes and Bayesian network Berrar, D. (2018). Bayes’ theorem and naive Bayes classifier. Encyclopedia of Bioinformatics and Computational Biology: ABC of Bioinformatics, 403, 412. K-nearest neighbor Peterson, L. E. (2009). K-nearest neighbor. Scholarpedia, 4(2), 1883.
	support vector machine Cortes, C., & Vapnik, V. (1995). Support-vector networks. Machine learning, 20, 273–297. Deep neural network Schmidhuber, J. (2015). Deep learning in neural networks: An overview. Neural networks, 61, 85–117.
Resource availability:	The code file is available.

## Method details

### Background

The broadband industry has experienced significant growth, as evidenced by data collected by the National Electronics and Computer Technology Center (NECTEC) on internet bandwidth in Thailand from 2016 to 2020 [1]. During this period, Thailand's international internet gateway bandwidth reached 14,144,148 Mbps, indicating a substantial increase of 30.61% compared to the previous year. This exponential growth has compelled broadband service providers to allocate substantial financial resources towards network development to keep up with the rapid advancements in technology and expand their coverage areas. Additionally, in order to compete with other providers, marketing strategies have been employed, resulting in a drastic reduction in service rates within the broadband industry over a relatively short span of time [2,4].

The cost of broadband services is primarily influenced by the devices utilized. Given the high costs associated with devices and the rapid pace of technological advancements, resources invested in previous devices may no longer be applicable today. With the increasing demand for bandwidth, device upgrades become a necessity. However, investing in network expansion to remote areas based on customer needs may not always be cost-effective.

Furthermore, in today's Internet-based economy, round-the-clock customer service is often a requirement. This necessitates highly available business networks that possess automated security measures to counter unexpected incidents. Additionally, these networks must be capable of adapting to varying traffic loads to ensure consistent application response times. The construction of networks by connecting standalone components without careful planning and design is no longer practical.

**Fig. 1:**
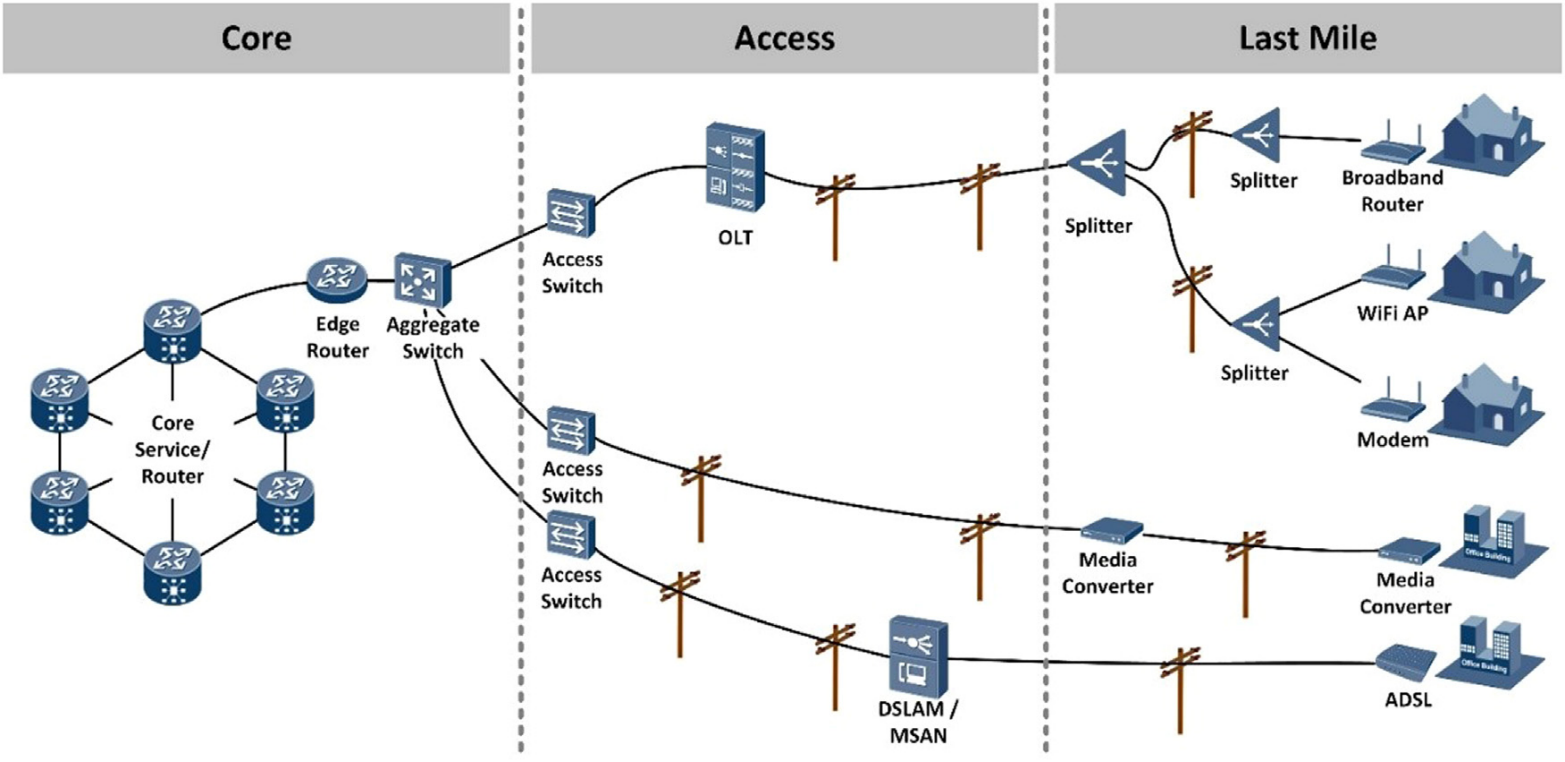
Broadband network. Core: Connects access layer devices. Access: Interconnects the smaller local networks. Last Mile: Provides connectivity for network hosts and end devices.

To fulfill fundamental design objectives, a network should be built upon an architecture that enables flexibility and scalability. In networking, a hierarchical design approach is commonly employed, organizing devices into multiple networks and employing a layered structure. The hierarchical design model typically encompasses three basic layers (Fig. 1) [3,5].

The process of recording newly purchased devices into the database is crucial for network planning, investment planning, maintenance planning, cost management, budget allocation, and analytical purposes within the broadband industry. However, the current procedure has limitations as engineers may not always provide immediate information regarding the specific network hierarchy to which a device belongs, leading to incomplete database records and hindering analytical capabilities [6].

In the field of text categorization, Thorsten Joachims [7] conducted a study on support vector machines (SVMs) and found that SVMs consistently outperformed existing methods in achieving good performance on categorization tasks. SVMs exhibit strong generalization capabilities in high-dimensional feature spaces, eliminating the need for feature selection and simplifying the application of text categorization. They also demonstrate robustness, avoiding catastrophic failures observed in conventional methods. Additionally, SVMs automatically find optimal parameter settings, further enhancing their usability as a method for learning text classifiers from examples.

Gary M. Weiss [8] explored data mining in the telecommunications industry and highlighted its significant applications in marketing, fraud detection, and network monitoring. The industry faces challenges due to the large-scale and sequential nature of the data, as well as real-time requirements. However, new methods have been developed and existing ones enhanced to address these challenges. The competitive and data-rich nature of the telecommunications industry emphasizes the importance of data mining for its future success.

Mishra BK et al. [9] investigated the benefits of data mining techniques and business intelligence in various industry sectors. Their findings revealed that industries such as banking, insurance, biomedical, customer management, and telecommunications derive competitive advantages through the utilization of data mining techniques. The integration of data mining with business intelligence systems provides efficient tools that support business operations effectively.

There are various works focused on the metaheuristic algorithms to solve specific problems such as space fractional advection-dispersion equation [10], the solute transport parameters [11], and crack detection of beam-type structure [12]. Drawing from the aforementioned works, the application of text mining techniques combined with the support vector machine classification algorithm presents a highly efficient tool. This approach enables real-time classification of data, previously requiring expert intervention, resulting in convenient and faster data classification. Furthermore, integrating data into business intelligence systems offers comprehensive support for business operations.

Considering the exponential growth of internet bandwidth in Thailand and the associated network investments, the classification of network devices becomes crucial for enhancing management efficiency in terms of network design, investment planning, maintenance planning, cost management [13], budget allocation, and further analytical purposes.

Therefore, this study presents a classification model for broadband network devices, leveraging text mining techniques and a classification algorithm, to enhance competitiveness within the industry. The major contributions and novel aspects of the work can be highlighted as follows:

1. We introduce a model that categorizes network devices to align with fundamental network design goals.
2. We proposed a novel model that predicts the replacement dates for network devices.
3. We offer a basic yet valuable approach to aid decision-making in network investment.
4. We introduce the capability to visualize large-scale data, facilitating better device management, maintenance planning, and budget allocation based on the network type.

## Methodology

**Fig. 2:**
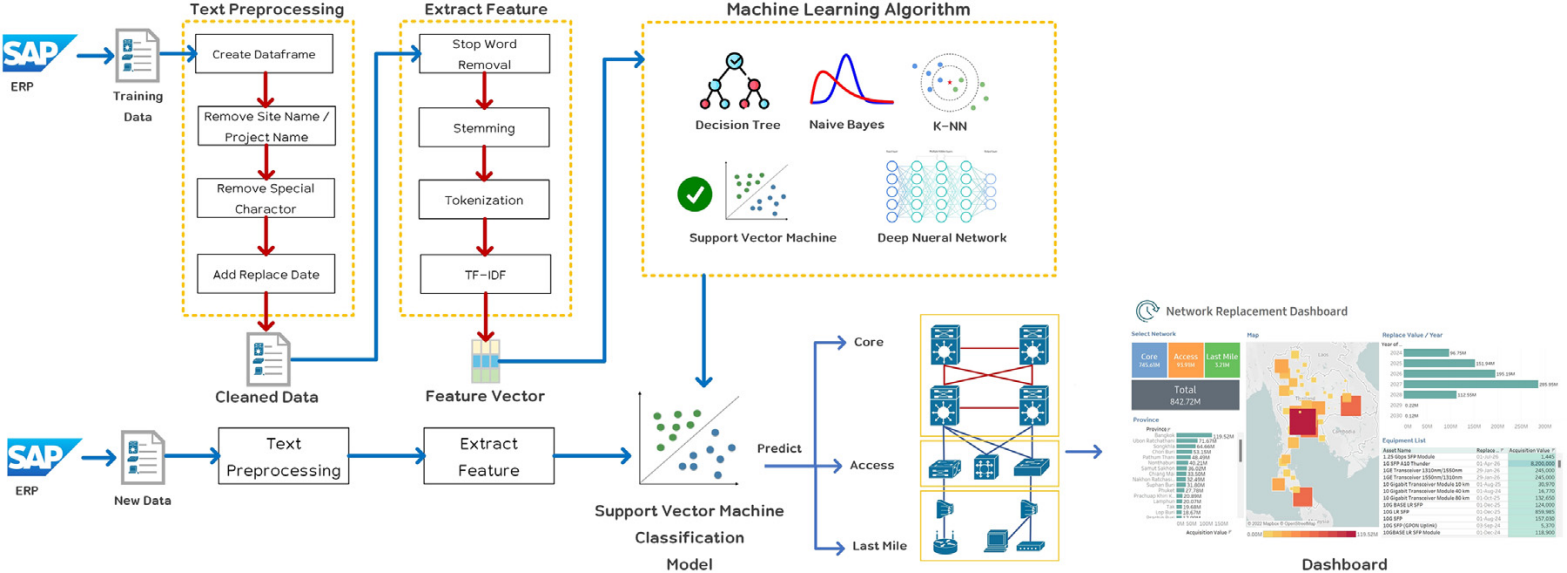
Conceptual Framework.

Fig. 2 presents the conceptual framework for the proposed method, which centers around the development of a model for classifying broadband devices into three distinct categories: core, access, and last mile. The classification process utilizes text mining methods in conjunction with classification algorithms to identify the most efficient algorithm for accurate device classification. Subsequently, the obtained results are aggregated and presented through visualization techniques.

The framework encompasses the following key steps:

1. Data Collection: Obtain a dataset comprising device information from a leading broadband network company in Thailand.
2. Preprocessing: Cleanse and preprocess the data by removing noise, standardizing formats, and performing necessary transformations.
3. Feature Extraction: Utilize text mining techniques to extract relevant features from the device descriptions, generating a feature vector representation for each device.
4. Classification Algorithms: Employ various classification algorithms such as decision tree, naïve Bayes, Bayesian network, k-nearest neighbor, support vector machine, and deep neural network to classify the devices into predefined categories.
5. Performance Evaluation: Compare the accuracy and performance of the classification algorithms to identify the most effective algorithm for accurate device classification.
6. Visualization: Display the classification results through visualization techniques, presenting information related to network hierarchy, device replacement dates, and budget requirements. This visual representation facilitates enhanced understanding and decision-making in areas such as cost management, budget planning, and investment decisions.

By following this conceptual framework, we aim to contribute to the efficient classification of broadband devices and provide valuable insights for network design, investment planning, maintenance planning, and budget allocation within the broadband industry.

### Training data

For our study, we gathered a dataset consisting of 100,419 broadband network equipment items sourced from one of Thailand's largest telecommunications companies. Each device entry in the dataset includes essential information such as department, province, device ID, description, network, acquisition details, and contract period (representing the device's expected lifetime as specified by the vendor).

### Text preprocessing

The text preprocessing procedure can be illustrated as the pseudocode as follows.

**Table:** 

***Procedure:****Text Preprocessing* ***Input:****Training Data* ***Output:****Cleaned Data*

*1****: Get****Training Data*
*2:* *For each row in Training Data****do***
*3:* ***Read****Description Column*
*4:* ***If****Description contains Thai characters*
*5:* ***then***
*6:* *Replace them with empty string*
*7:* ***If****Description contains Special characters*
*8:* ***then***
*9:* *Replace them with empty string*
*10:* *For each row in Training Data****do***
*11:* ***Insert****Replace Date Column*
*12:* ***Read****Acquisition Date Column*
*13:* ***Read****Contract Period Column*
*14:* *Replace Date = Acquisition Date + Contract Period* *15* ***Calculate****Replace Date*
*16:* ***Write****Replace Date to Replace Date Column*
*17:****Export****Cleaned Data*

During the Text Preprocessing phase, we executed the following steps:

**1. Removal of sitename/project name:**

In this step, we eliminated Thai letters within the description column that referred to Sitename or Project Name. The aim was to retain only the device's name and type within the description field while removing any extraneous information.

**Table:** 

For each row in Training Data
Read Description Column
If Description contains Thai characters
Then
Replace them with empty string

**2. Removal of special characters:**

To further refine the text data, we performed the removal of special characters from the description column. Special characters, including but not limited to (), {}, [],:,;, #, *, ", ', /, \, |, +, and -, were eliminated from the description column. This process ensured that the text only contained relevant information for subsequent analysis and classification tasks.

**Table:** 

For each row in Training Data
Read Description Column
If Description contains Special characters
then
Replace them with empty string

**3. Add replacement date**

In order to include the replacement date information, we introduced a new column named "Replacement Date" in the dataframe. This column was created by adding the contract period to the acquisition date for each device entry. By combining these values, we obtained an estimated replacement date that signifies when the device is anticipated to require replacement. This addition enhances the dataframe by providing valuable insights into the expected lifespan of each device, facilitating effective planning and management of device replacements within the broadband network system.

**Table:** 

For each row in Training Data Insert Replace Date Column Read Acquisition Date Column Read Contract Period Column Replace Date = Acquisition Date + Contract Period Calculate Replace Date Write Replace Date to Replace Date Column

### Feature extraction

The feature extraction process can be illustrated as the pseudocode as follows.

**Table:** 

***Procedure:****Extract Feature* ***Input:****Cleaned Data* ***Output:****Feature Vector*

*1****: Get****Cleaned Data*
*2:* *For each row in Cleaned Data****do***
*3:* ***Read****Description Column*
*4:* ***If****Description contains Stop Word*
*5:* ***then***
*6:* *Remove them*
*7:* ***If****Description contains Suffixed*
*8:* ***then***
*9:* *Replace them with root word*
*10:* *For each row in Training Data****do***
*11:* ***Use****Tokenize* *12:* *TF-IDF =*$tf_{i,j}\;\times \;\text{log}\left(\frac{N}{df_{i\;}}\right)$
*13:* ***Calculate****TF-IDF (Feature Vector)*
*14:****Export****Feature Vector*

The detail of feature extraction procedure can be described as:

1. Stop Word Removal:

To reduce the dimensionality of the feature vector, we implemented the technique of removing stop words. These are commonly occurring words in a language that do not carry significant meaning or contribute much to the overall understanding of the text. By eliminating stop words, we aimed to streamline the feature vector while ensuring that the essential information and context of the text were retained.

2. Stemming:

Then, we employed stemming to further reduce the feature vector's size and increase its efficiency. This technique involves reducing words to their root form by removing any suffixes or prefixes. By converting words to their base or root forms, we aimed to consolidate similar words with similar meanings into a single representative term. This process helps to eliminate redundancy and improve the overall effectiveness of the feature vector.

3. Tokenization:

After that we utilized tokenization in order to generate feature vectors. This technique involves segmenting the text into individual words or tokens by using spaces as separators. Each word obtained from the tokenization process was then considered as an attribute within the dataset. Tokenization facilitates the conversion of the textual information into a structured format that can be utilized for subsequent analysis and classification tasks. By implementing these feature extraction techniques, we aimed to enhance the efficiency and effectiveness of the classification model by reducing the dimensionality of the feature vector while preserving the essential information needed for accurate device classification.

4. TF-IDF

Then we computed the weight using Term Frequency-Inverse Document Frequency (TF-IDF) technique, a statistical measure that evaluates how relevant a word is to a document in a collection of documents. TF-IDF is a technique of data modeling that computes a weight for each word which indicates the importance of the word for a given document within a corpus. TF defines the occurrence of a word *w* in a document *d*. IDF measures the rarity of a word *w* in the whole document. The weight of TF-IDF was calculated from the following equation:

(1) $$\omega_{i,j}=\;tf_{i,j}\;\times \;\text{log}\left(\frac{N}{df_{i\;}}\right)$$

where:

$tf_{i,j}$= number of occurrences of *i* in *j*
$df_{i\;}$= number of documents containing *i*
N = total number of documents

### Classification

We develop the classification model using Support Vector Machine [14], [15], [16], [17], [18], [19], [20], [21] to devise a computationally efficient way of learning good separating hyperplanes in a high dimensional feature space. In the following, the construction of such a hyperplane is described using the Maximum Margin Classifier as an example of a linear machine.

In our implementation, we construct a hyperplane using the Maximum Margin Classifier, which serves as an example of a linear machine. Let *τ* = {(${\overset{⇀}{x}}_{i}+$ *y_i_*)}; *i* = 1, …, *k*; ${\overset{⇀}{x}}_{i}+$ ∈ $R^{n}$; *y_i_*) ∈ {−1, +1} be a linearly separable training set. The feature vectors (${\overset{⇀}{\omega }}^{T}\overset{⇀}{x}+b$) obtained from the feature extraction process are used in the SVM classification. We aim to find a hyperplane that separates the positive (*y_i_ =* +1) from the negative (*y_i_* = −1) training examples. The hyperplane can be represented as shown in Eq. (2):

(2) $${\overset{⇀}{\omega }}^{T}\overset{⇀}{x}+b=0$$

$${\overset{⇀}{\omega }}^{T}{\overset{⇀}{x}}_{i}+b\geq 0\;for\;y_{i}=\;+1,$$

$${\overset{⇀}{\omega }}^{T}{\overset{⇀}{x}}_{i}+b\;<0\;for\;y_{i}=\;-1,$$

where $\overset{⇀}{\omega }$ is the normal to the hyperplane and b is the perpendicular distance of the hyperplane to the origin. A decision function is shown in Eq. (3).

(3) $$g\left(\overset{⇀}{x}\right)=\;{\overset{⇀}{\omega }}^{T}{\overset{⇀}{x}}_{i}+b$$

Therefore, can be interpreted as the functional distance of an instance from the hyperplane. For $g(\overset{⇀}{x})<0,$ the instance would be classified negative as it lies below the decision surface, and it would be classified positive if $g(\overset{⇀}{x})\geq 0$ as it lies on or above the surface.

One reason for the success of SVM is its ability to effectively classify data with complex boundaries. SVM uses a technique called kernel trick, which transforms data into a higher dimensional space where it can be more easily separated. This allows SVM to accurately classify data that would be difficult or impossible for other algorithms. Additionally, SVM has a regularizing effect, which helps prevent overfitting and improves generalization on new data. Overall, SVM's combination of powerful classification capabilities and effective regularization make it a popular and successful machine learning algorithm.

The goal of SVM is to maximize the margin between the positive and negative training examples while minimizing classification errors. By finding the optimal hyperplane, we aim to achieve the best separation between the two classes, allowing for the accurate classification of new, unseen data points. The SVM algorithm leverages the concept of support vectors, which are the data points closest to the decision boundary. These support vectors play a crucial role in defining the hyperplane and determining the classification boundaries.

Through the utilization of the SVM algorithm, we aim to develop a robust and efficient classification model that can accurately classify broadband network devices into the appropriate categories, namely core, access, and last mile. However, it is important to note that in many real-world scenarios, the requirement of linear separability of data points is not always met. In such cases, the "kernel trick" is employed. This method involves transforming the input data into a higher-dimensional space in a way that makes it linearly separable (Table 1). By applying the kernel trick, we can effectively handle complex patterns and non-linear relationships within the data. The transformed feature vectors enable the SVM algorithm to find optimal separating hyperplanes in the higher-dimensional space, even if the original data was not linearly separable.

**Table 1:** Inner-Product Kernels.

Kernel	Inner Product Kernel $K(\overset{⇀}{x},{\overset{⇀}{x}}_{i}),\;i=1,2,\ldots ,N$
Linear Kernel	$K\left(\overset{⇀}{x},{\overset{⇀}{x}}_{i}\right)={\overset{⇀}{x}}^{T}{\overset{⇀}{x}}_{i}$
Second Polynomial Kernel	$K\left(\overset{⇀}{x},{\overset{⇀}{x}}_{i}\right)={\left({\overset{⇀}{x}}^{T}{\overset{⇀}{x}}_{i}+\theta \right)}^{2}$
Third Polynomial Kernel	$K\left(\overset{⇀}{x},{\overset{⇀}{x}}_{i}\right)={\left({\overset{⇀}{x}}^{T}{\overset{⇀}{x}}_{i}+\theta \right)}^{3}$
RBF Kernel	$K\left(\overset{⇀}{x},{\overset{⇀}{x}}_{i}\right)=\;e^{-\gamma {||\overset{⇀}{x}-{\overset{⇀}{x}}_{i}||}^{2}}$
Sigmoid Kernel	$K\left(\overset{⇀}{x},{\overset{⇀}{x}}_{i}\right)=tanh\left(\eta \overset{⇀}{x}{\overset{⇀}{x}}_{i}+\theta \right)$

### Model evaluation

**Fig. 3:**
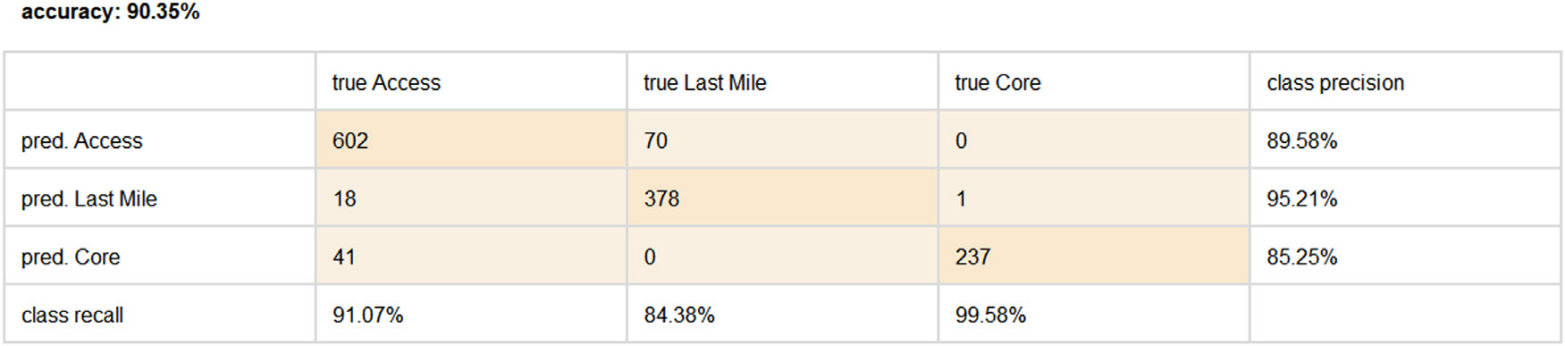
Confusion Matrix and Performance Metrics of the classification model.

To assess the performance of our classification model, we employ the use of a confusion matrix. The confusion matrix allows us to calculate various performance metrics that provide valuable insights into the accuracy and effectiveness of the model (Table 2 and Fig. 3).

**Table 2:** Confusion Matrix and Performance Metrics.

Derivations	Equation
Precision	$\frac{TP}{TP+FP}$
Recall	$\frac{TP}{TP+FN}$
Accuracy	$\frac{TP+TN}{TP+FN+FP+FN}$
F1 Score	$\frac{2\;\times \;Precision\;\times \;Recall}{Precesion+Recall}$

In the confusion matrix, the rows represent the actual classes of the broadband network devices, while the columns represent the predicted classes. The diagonal elements of the matrix represent the true positives (TP), where the predicted and actual classes match correctly. The off-diagonal elements indicate the false positives (FP) and false negatives (FN), where the predicted and actual classes do not match.

Based on the values in the confusion matrix, we can calculate the following performance metrics:

- Accuracy: The overall accuracy of the model, which measures the proportion of correctly classified instances.
- Precision: The precision of each class, indicating the ability of the model to correctly identify positive instances.
- Recall: The recall of each class, representing the proportion of true positives identified by the model.
- F1-score: A weighted average of precision and recall, providing a balanced measure of the model's performance.

These performance metrics provide a comprehensive evaluation of the classification model's effectiveness in accurately categorizing broadband network devices into their respective classes. By analyzing these metrics, we can gain valuable insights into the model's performance and make informed decisions regarding network design, investment planning, maintenance, cost management, and other analytical purposes.

## Method validation

In this work, textual content from the training dataset underwent a text analysis, leveraging the TF-IDF method to formulate feature vectors. This analysis produced 4683 attributes for the dataset. For the experimental trials, the dataset was segmented into 70% training and 30% testing portions. Several classification algorithms were employed, namely: decision tree, naïve Bayes, Bayesian network, k-nearest neighbor, support vector machine, and deep neural network. The efficiency of these algorithms was gauged based on metrics like precision, recall, accuracy, and F1 score.

Upon evaluation, it was discerned that the support vector machine, particularly with the utilization of the second polynomial kernel, rendered the most superior classification outcomes. This algorithm recorded an accuracy rate of 90.35%, a precision rate of 90.01%, a recall rate of 91.68%, and an F1 score of 90.84% (Table 3).

**Table 3:** Model evaluation results.

Algorithms	Conditions	Accuracy	Precision	Recall	F1 Score
Decision Tree	Max. Dept: 5	43.43	33.30	52.65	40.80
	Max. Dept: 10	45.14	34.72	55.87	42.83
	Max. Dept: 15	46.47	35.62	58.39	44.25
	Max. Dept: 20	44.39	33.46	55.46	41.74
	Max. Dept: 25	44.39	33.46	55.46	41.74
Naïve Bayes	Estimation Mode: Full	74.61	73.93	80.84	77.23
Bayesian Networks	Simple Estimator	67.85	69.51	72.53	70.99
K-Nearest Neighbors	*K* = 1	78.99	78.62	82.40	80.47
	*K* = 2	79.14	78.73	82.55	80.59
	*K* = 3	76.17	75.25	80.87	77.96
	*K* = 4	76.24	75.33	80.92	78.03
	*K* = 5	74.76	73.83	79.89	76.74
Support Vector Machine (C = 0 Epsilon = 0.001 Gamma = 0)	Linear Kernel	89.68	88.45	92.13	90.25
	Second Polynomial Kernel	90.35	90.01	91.68	90.84
	Third Polynomial Kernel	83.25	86.69	84.62	85.64
	RBF Kernel	33.26	11.09	33.33	16.64
	Sigmoid Kernel	89.68	88.45	92.13	90.25
Deep Neural Network (Epoch = 100 Learning Rate = 0.01, Hidden Layer = 3, Neural = 50)	ReLU	83.67	82.12	87.76	84.85
	Sigmoid	81.66	80.17	86.35	83.15
	Softmax	81.74	80.52	86.14	83.24
	TanH	82.55	81.01	86.91	83.86

Table 3 shows the details of the model evaluation and hyperparameter setting. We use three hidden layers in our deep neural network because the more hidden layers, the model becomes more complex while does not provide higher accuracy. Since the amount of available data in our work is limited, adding more layers may lead to overfitting. By choosing three layers, we have been trying to find a balance between the model's capacity and the risk of overfitting. Additionally, fewer hidden layers would also not contribute to the performance of our model. Thus, in this work, we choose three hidden layers as they provide the best performance in terms of accuracy. It is also a compromise between the depth and feasibility of the hardware as well.

### Interactive dashboard

**Fig. 4:**
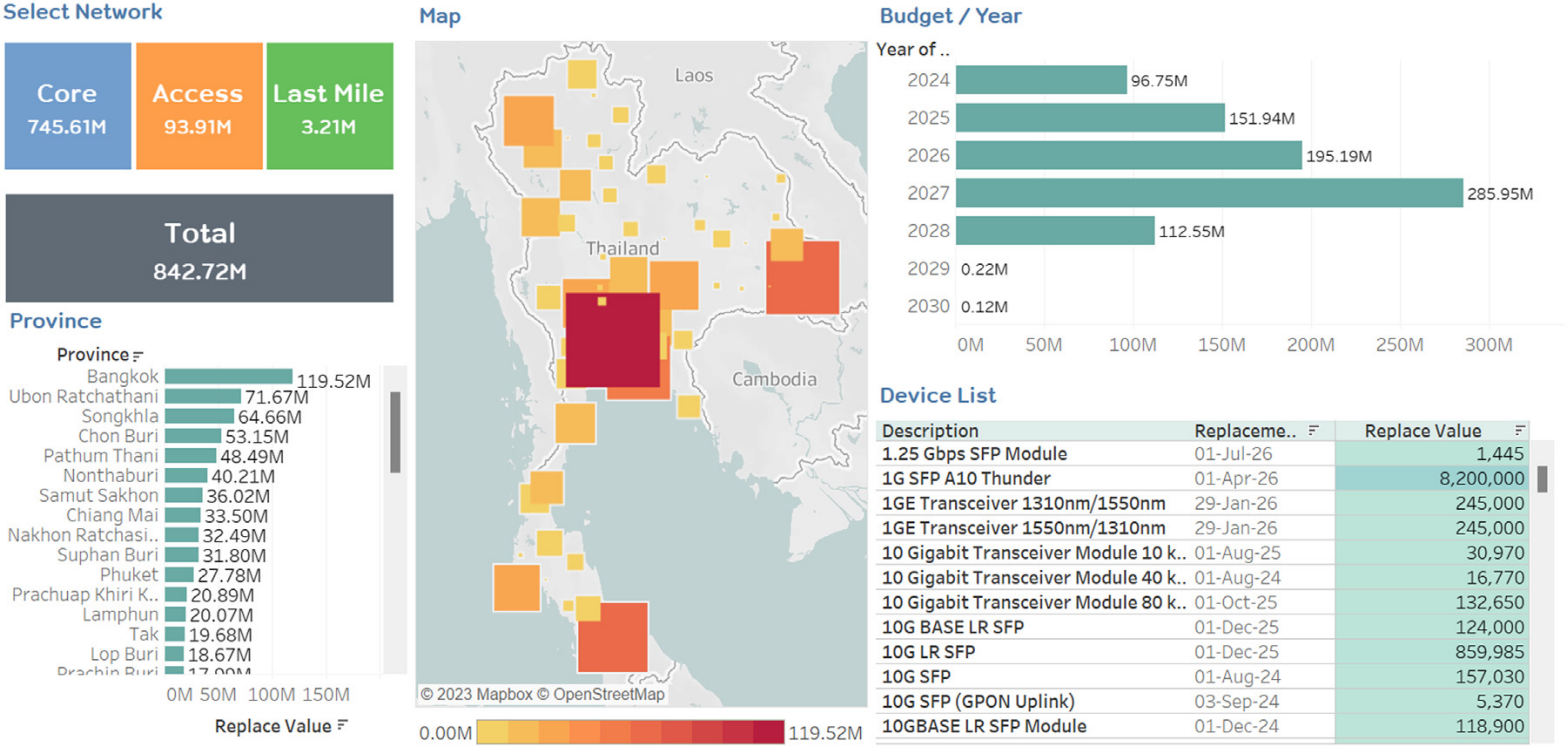
Data visualization screen.

This work includes the development of an interactive dashboard that presents the information obtained from the classification model [22] as shown in Fig. 4. This dashboard provides valuable insights for device management and budget allocation based on the network type. It consists of five sections:

1. Select Network: This section allows users to choose the network type, such as core, access, or last mile. The other sections of the dashboard will dynamically display information based on the selected network type.
2. Province: This section displays the budget required for device replacements in each province, sorted in descending order. Users can easily see the provinces that require the highest budgets for device replacements.
3. Map: This section presents a map of Thailand, where the size and color of the squares vary based on the budget required in each province. Provinces with higher budget requirements will be represented by larger and darker squares, while provinces with lower requirements will have smaller and lighter squares. This visual representation allows users to quickly grasp the budget requirements across different provinces.
4. Budget/Year: This section presents the budget required for device replacements each year in the form of a bar chart. Users can analyze the budget trends over time and identify any significant variations or patterns.
5. Device List: This section displays a list of devices based on the selections made in other sections. It provides information such as device names, replacement dates, and their corresponding values. Users can easily access specific device information according to their interests.

### Browse data by network type

Users can select the network type from the "Select Network" section. Upon selection, the other sections of the dashboard will display information specific to the chosen network type. For example, selecting "access" will show the budget required for access network replacements in the province section, highlight the provinces needing replacements on the map, present the budget required for access network replacements in the budget/year section, and list the devices and their replacement dates specific to the access network in the device list section.

### Browse data by province

Users can select a specific province from the "Province" section to obtain information relevant to that province. The other sections of the dashboard will update accordingly. For instance, selecting "Phuket" will display the budget requirements for each network segment (core, access, last mile) specific to Phuket in the "Select Network" section, show a heatmap of Phuket in the map section, present the annual budget required for device replacements in Phuket in the budget/year section, and list the devices and their replacement dates for Phuket in the device list section.

### Browse data by budget/year

Users can select a particular year from the "Budget/Year" section to explore the budget required for device replacements in that specific year. The other sections of the dashboard will update accordingly. For example, selecting "2025″ will show the budget requirements for each network type (core, access, last mile) in the "Select Network" section, highlight the provinces needing replacements in 2025 on the map, list the provinces requiring replacements in 2025 in the province section, and display the device list that needs replacements in 2025 in the device list section.

The interactive dashboard provides users with a user-friendly and informative interface to explore and analyze the classification model's results. It facilitates decision-making processes related to device management, budget allocation, and network planning in the broadband industry.

## Discussion

In the subsequent discussion, we delve deeper into the pivotal findings and insights stemming from our work, addressing the intricacies of our proposed model and its practical implications.

### Model performance and feature extraction with TF-IDF

The core of our research focused on devising a model for broadband network device classification, harnessing the powers of text-mining techniques combined with adept classification algorithms. A salient observation was that the integration of the TF-IDF method for feature extraction, in tandem with the support vector machine algorithm, culminated in an exceptionally effective model. Achieving an accuracy rate of 90.35% is a testament to the model's robustness in categorizing broadband network devices with precision. One cornerstone underpinning this success was undoubtedly the TF-IDF method. It distinguished itself in its capacity to derive feature vectors from device descriptions. Its efficacy lay in its ability to accentuate terms pivotal to specific documents, thereby enhancing the classification precision. By doing so, the TF-IDF method aptly encapsulated relevant information, facilitating the differentiation of diverse device categories.

### Role of support vector machine

Complementing the achievements of the TF-IDF method was the support vector machine's stellar performance. It was singled out primarily due to its adeptness at discerning efficient separating hyperplanes within expansive, high-dimensional feature arenas. Our experimentation revealed that when paired with a second polynomial kernel, this algorithm not only excelled in accuracy but also exhibited commendable processing speeds. Such attributes underscore the support vector machine's adaptability and relevance to text classification endeavors, especially within the broadband network device milieu.

### Dataset challenges

While our results were promising, it's imperative to shed light on challenges encountered, particularly with the dataset. We discerned minor inaccuracies within the device descriptions, manifesting as typographical errors and sporadic whitespace inconsistencies. Nevertheless, these discrepancies were overshadowed by the overarching dataset and, thankfully, did not impart any significant adversities to the classification mechanism. To elevate future models, there's an inherent need to ensure impeccable data quality and maintain uniformity.

Conclusively, our revelations underline the prowess of our proposed model, which seamlessly amalgamates text-mining techniques with the support vector machine algorithm, setting a new benchmark in broadband network device classification. These experimental outcomes not only echo the model's potential but also spotlight its prospective applications in optimizing device management and financial planning within the telecommunications sphere. For forthcoming research endeavors, the expansion of the dataset and the incorporation of cutting-edge techniques might pave the way for even more refined classification proficiencies.

## Conclusion

In conclusion, we developed a model for classifying broadband network devices using text mining techniques and a classification algorithm. The model demonstrated high accuracy in categorizing devices into core, access, and last mile types. The TF-IDF technique was effective in extracting relevant features from the device descriptions, and the support vector machine algorithm yielded the best classification results. The visualization of the results through an interactive dashboard provided valuable information for device management and budget allocation. Users can easily explore data based on network types, provinces, and budget years, enabling informed decision-making and efficient analysis. Overall, this work contributes to the field of broadband network device classification and demonstrates the potential of text mining and visualization techniques in enhancing decision-making processes in the telecommunications industry. Future work could involve incorporating network utilization data to enhance investment decisions. By considering the capacity used in each network and province, more accurate insights can be gained, leading to improved resource allocation and optimization.

## Ethics statements

This work is not involved with human subjects, animal experiments, and data collected from social media platforms.

## CRediT authorship contribution statement

**Mahasak Ketcham:** Conceptualization, Methodology, Validation, Writing – original draft. **Thittaporn Ganokratanaa:** Software, Investigation, Writing – review & editing. **Nattapat Sridoung:** Conceptualization, Methodology, Visualization.

## Declaration of Competing Interest

The authors declare that they have no known competing financial interests or personal relationships that could have appeared to influence the work reported in this paper.

## Data Availability

Data will be made available on request.

## Appendix Supplementary materials

A dataset is available on request.

Image, application 1
