# Supplementary material for: Classification of broadband network devices using text mining technique
Source: MethodsX. 2023 Aug 30;11:102346. doi: 10.1016/j.mex.2023.102346 (PMC10477059; doi:10.1016/j.mex.2023.102346)
Supplement: Supplementary file 1 [file mmc1.docx]

Python code

import pandas as pd

from google.colab import drive

drive.mount('/gdrive')

%cd /gdrive

df = pd.read_csv('/gdrive/MyDrive/Colab Notebooks/Training_Data_2023.csv')

df['Description'] = df['Description'].str.replace('\W+',' ')

df['Description'] = df['Description'].str.replace('[กขคฅฆงจฉชซฌญฎฏฐฑฒณดตถทธนบปผฝพฟภมยรฤลฦวศษสหฬอฮะ◌ัาำ◌ิ◌ี◌ึ◌ื◌ุ◌ู◌ฺ฿เแโใไๅๆ◌็◌่◌้◌๊◌๋◌์◌ํ◌๎๏๐๑๒๓๔๕๖๗๘๙๚ฯ๛]','')

df['Replace Date'] = pd.to_datetime(df['Acquisition Date']) + pd.to_timedelta(df['Contract'] * 365, unit='D')

df.to_csv(r'/gdrive/MyDrive/Colab Notebooks/Clean_Data_02_Jan_2023.csv', index = False)

RapidMiner Diagram


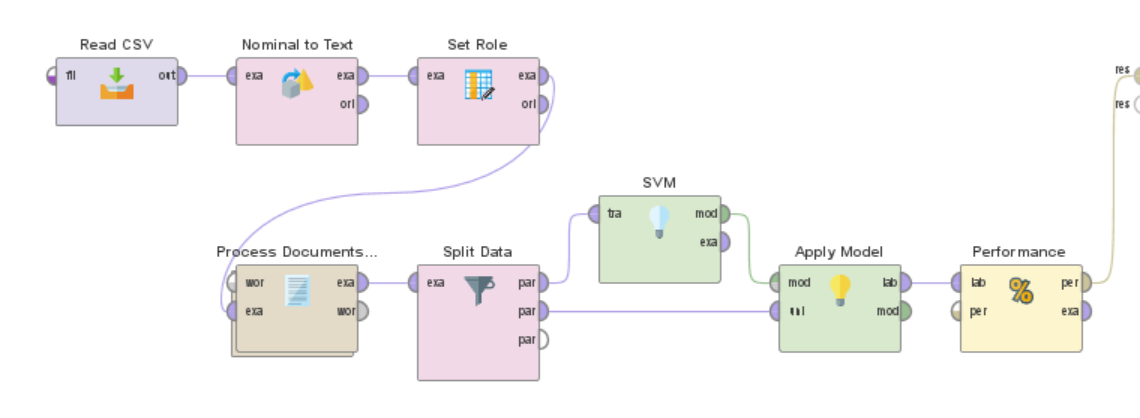


Process Document


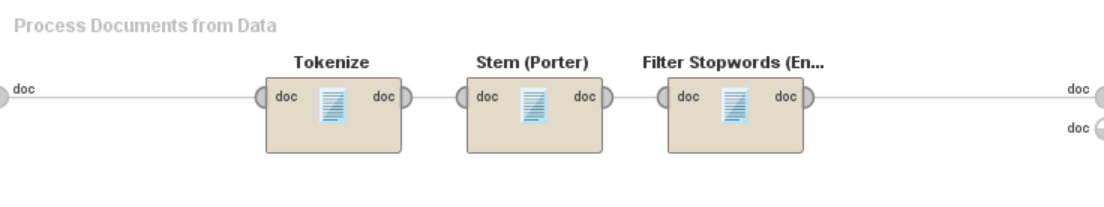


XML Code from Rapidminer

| <process version="9.10.010">  <context>  <input/>  <output/>  <macros/>  </context>  <operator activated="true" class="process" compatibility="9.10.010" expanded="true" name="Process">  <parameter key="logverbosity" value="init"/>  <parameter key="random_seed" value="2001"/>  <parameter key="send_mail" value="never"/>  <parameter key="notification_email" value=""/>  <parameter key="process_duration_for_mail" value="30"/>  <parameter key="encoding" value="SYSTEM"/>  <process expanded="true">  <operator activated="true" class="read_csv" compatibility="9.10.010" expanded="true" height="68" name="Read CSV" width="90" x="1117" y="34">  <parameter key="csv_file" value="D:\Google Drive\MIS\IS\Training_Data_Cleansing.csv"/>  <parameter key="column_separators" value=","/>  <parameter key="trim_lines" value="false"/>  <parameter key="use_quotes" value="true"/>  <parameter key="quotes_character" value="""/>  <parameter key="escape_character" value="\"/>  <parameter key="skip_comments" value="true"/>  <parameter key="comment_characters" value="#"/>  <parameter key="starting_row" value="1"/>  <parameter key="parse_numbers" value="true"/>  <parameter key="decimal_character" value="."/>  <parameter key="grouped_digits" value="false"/>  <parameter key="grouping_character" value=","/>  <parameter key="infinity_representation" value=""/>  <parameter key="date_format" value=""/>  <parameter key="first_row_as_names" value="true"/>  <list key="annotations"/>  <parameter key="time_zone" value="SYSTEM"/>  <parameter key="locale" value="English (United States)"/>  <parameter key="encoding" value="x-windows-874"/>  <parameter key="read_all_values_as_polynominal" value="false"/>  <list key="data_set_meta_data_information">  <parameter key="0" value="ASSET_NAME.true.polynominal.attribute"/>  <parameter key="1" value="NETWORK.true.polynominal.attribute"/>  </list>  <parameter key="read_not_matching_values_as_missings" value="false"/>  </operator>  <operator activated="true" class="nominal_to_text" compatibility="9.10.010" expanded="true" height="82" name="Nominal to Text" width="90" x="1251" y="34">  <parameter key="attribute_filter_type" value="single"/>  <parameter key="attribute" value="ASSET_NAME"/>  <parameter key="attributes" value=""/>  <parameter key="use_except_expression" value="false"/>  <parameter key="value_type" value="nominal"/>  <parameter key="use_value_type_exception" value="false"/>  <parameter key="except_value_type" value="file_path"/>  <parameter key="block_type" value="single_value"/>  <parameter key="use_block_type_exception" value="false"/>  <parameter key="except_block_type" value="single_value"/>  <parameter key="invert_selection" value="false"/>  <parameter key="include_special_attributes" value="false"/>  </operator>  <operator activated="true" class="set_role" compatibility="9.10.010" expanded="true" height="82" name="Set Role" width="90" x="1385" y="34">  <parameter key="attribute_name" value="NETWORK"/>  <parameter key="target_role" value="label"/>  <list key="set_additional_roles"/>  </operator>  <operator activated="true" class="text:process_document_from_data" compatibility="9.4.000" expanded="true" height="82" name="Process Documents from Data" width="90" x="1251" y="187">  <parameter key="create_word_vector" value="true"/>  <parameter key="vector_creation" value="TF-IDF"/>  <parameter key="add_meta_information" value="true"/>  <parameter key="keep_text" value="false"/>  <parameter key="prune_method" value="none"/>  <parameter key="prune_below_percent" value="3.0"/>  <parameter key="prune_above_percent" value="30.0"/>  <parameter key="prune_below_rank" value="0.05"/>  <parameter key="prune_above_rank" value="0.95"/>  <parameter key="datamanagement" value="double_sparse_array"/>  <parameter key="data_management" value="auto"/>  <parameter key="select_attributes_and_weights" value="false"/>  <list key="specify_weights"/>  <process expanded="true">  <operator activated="true" class="text:tokenize" compatibility="9.4.000" expanded="true" height="68" name="Tokenize" width="90" x="179" y="34">  <parameter key="mode" value="non letters"/>  <parameter key="characters" value=" "/>  <parameter key="language" value="English"/>  <parameter key="max_token_length" value="3"/>  </operator>  <operator activated="true" class="text:stem_porter" compatibility="9.4.000" expanded="true" height="68" name="Stem (Porter)" width="90" x="313" y="34"/>  <operator activated="true" class="text:filter_stopwords_english" compatibility="9.4.000" expanded="true" height="68" name="Filter Stopwords (English)" width="90" x="447" y="34"/>  <connect from_port="document" to_op="Tokenize" to_port="document"/>  <connect from_op="Tokenize" from_port="document" to_op="Stem (Porter)" to_port="document"/>  <connect from_op="Stem (Porter)" from_port="document" to_op="Filter Stopwords (English)" to_port="document"/>  <connect from_op="Filter Stopwords (English)" from_port="document" to_port="document 1"/>  <portSpacing port="source_document" spacing="0"/>  <portSpacing port="sink_document 1" spacing="0"/>  <portSpacing port="sink_document 2" spacing="0"/>  </process>  </operator>  <operator activated="true" class="split_data" compatibility="9.10.010" expanded="true" height="103" name="Split Data" width="90" x="1385" y="187">  <enumeration key="partitions">  <parameter key="ratio" value="0.7"/>  <parameter key="ratio" value="0.3"/>  </enumeration>  <parameter key="sampling_type" value="automatic"/>  <parameter key="use_local_random_seed" value="false"/>  <parameter key="local_random_seed" value="1992"/>  </operator>  <operator activated="true" class="support_vector_machine_libsvm" compatibility="9.10.010" expanded="true" height="82" name="SVM" width="90" x="1519" y="136">  <parameter key="svm_type" value="C-SVC"/>  <parameter key="kernel_type" value="poly"/>  <parameter key="degree" value="2"/>  <parameter key="gamma" value="0.0"/>  <parameter key="coef0" value="0.0"/>  <parameter key="C" value="0.0"/>  <parameter key="nu" value="0.5"/>  <parameter key="cache_size" value="80"/>  <parameter key="epsilon" value="0.001"/>  <parameter key="p" value="0.1"/>  <list key="class_weights"/>  <parameter key="shrinking" value="true"/>  <parameter key="calculate_confidences" value="false"/>  <parameter key="confidence_for_multiclass" value="true"/>  </operator>  <operator activated="true" class="apply_model" compatibility="9.10.010" expanded="true" height="82" name="Apply Model" width="90" x="1653" y="187">  <list key="application_parameters"/>  <parameter key="create_view" value="false"/>  </operator>  <operator activated="true" class="performance_classification" compatibility="9.10.010" expanded="true" height="82" name="Performance" width="90" x="1787" y="187">  <parameter key="main_criterion" value="first"/>  <parameter key="accuracy" value="true"/>  <parameter key="classification_error" value="false"/>  <parameter key="kappa" value="false"/>  <parameter key="weighted_mean_recall" value="false"/>  <parameter key="weighted_mean_precision" value="false"/>  <parameter key="spearman_rho" value="false"/>  <parameter key="kendall_tau" value="false"/>  <parameter key="absolute_error" value="false"/>  <parameter key="relative_error" value="false"/>  <parameter key="relative_error_lenient" value="false"/>  <parameter key="relative_error_strict" value="false"/>  <parameter key="normalized_absolute_error" value="false"/>  <parameter key="root_mean_squared_error" value="false"/>  <parameter key="root_relative_squared_error" value="false"/>  <parameter key="squared_error" value="false"/>  <parameter key="correlation" value="false"/>  <parameter key="squared_correlation" value="false"/>  <parameter key="cross-entropy" value="false"/>  <parameter key="margin" value="false"/>  <parameter key="soft_margin_loss" value="false"/>  <parameter key="logistic_loss" value="false"/>  <parameter key="skip_undefined_labels" value="true"/>  <parameter key="use_example_weights" value="true"/>  <list key="class_weights"/>  </operator>  <connect from_op="Read CSV" from_port="output" to_op="Nominal to Text" to_port="example set input"/>  <connect from_op="Nominal to Text" from_port="example set output" to_op="Set Role" to_port="example set input"/>  <connect from_op="Set Role" from_port="example set output" to_op="Process Documents from Data" to_port="example set"/>  <connect from_op="Process Documents from Data" from_port="example set" to_op="Split Data" to_port="example set"/>  <connect from_op="Split Data" from_port="partition 1" to_op="SVM" to_port="training set"/>  <connect from_op="Split Data" from_port="partition 2" to_op="Apply Model" to_port="unlabelled data"/>  <connect from_op="SVM" from_port="model" to_op="Apply Model" to_port="model"/>  <connect from_op="Apply Model" from_port="labelled data" to_op="Performance" to_port="labelled data"/>  <connect from_op="Performance" from_port="performance" to_port="result 1"/>  <portSpacing port="source_input 1" spacing="0"/>  <portSpacing port="sink_result 1" spacing="0"/>  <portSpacing port="sink_result 2" spacing="0"/>  </process>  </operator>  </process> |
| --- |
